# Applications of Computer Vision-Based Structural Monitoring on Long-Span Bridges in Turkey

**DOI:** 10.3390/s23198161

**Published:** 2023-09-29

**Authors:** Chuanzhi Dong, Selcuk Bas, Fikret Necati Catbas

**Affiliations:** 1 Department of Civil, Environmental, and Construction Engineering, University of Central Florida, Orlando, FL 32816, USA; ceczdong@knights.ucf.edu (C.D.); sbas@bartin.edu.tr (S.B.); 2 Department of Civil Engineering, Bartin University, Bartin 74110, Turkey

**Keywords:** long-span bridges, computer vision, displacement, structural health monitoring

## Abstract

Structural displacement monitoring is one of the major tasks of structural health monitoring and it is a significant challenge for research and engineering practices relating to large-scale civil structures. While computer vision-based structural monitoring has gained traction, current practices largely focus on laboratory experiments, small-scale structures, or close-range applications. This paper demonstrates its applications on three landmark long-span suspension bridges in Turkey: the First Bosphorus Bridge, the Second Bosphorus Bridge, and the Osman Gazi Bridge, among the longest landmark bridges in the world, with main spans of 1074 m, 1090 m, and 1550 m, respectively. The presented studies achieved non-contact displacement monitoring from a distance of 600 m, 755 m, and 1350 m for the respective bridges. The presented concepts, analysis, and results provide an overview of long-span bridge monitoring using computer vision-based monitoring. The results are assessed with conventional monitoring approaches and finite element analysis based on observed traffic conditions. Both displacements and dynamic frequencies align well with these conventional techniques and finite element analyses. This study also highlights the challenges of computer vision-based structural monitoring of long-span bridges and presents considerations such as the encountered adverse environmental factors, target and algorithm selection, and potential directions of future studies.

## 1. Introduction

### 1.1. Statement of the Problem

Long-span bridges serve as critical links in transportation networks and are significant components of civil infrastructure systems in terms of functionality, serviceability, and safety [1,2,3]. The investment required for building long-span bridges and the complexity of the structures are much higher than other types of bridges. Long-span bridges are constructed to provide passage across large waterways such as rivers, channels, and seas [4]. Due to adverse environmental effects and external loads, such as temperature cycles, chloride ion invasion, scouring, increasing traffic loads, and occasional impact loads or collisions, etc., bridges show effects of deterioration over time and become damaged. Bridge aging, gradual degradation of structural materials and components, and unexpected extreme events could hasten the reduction in bridge performance and lead to early functional intervention, load permit post, and costly replacement, which waste resources and harm the development of sustainable societies. For long-span bridges, the large size poses challenges of inspection, maintenance, and management, and highly increases the cost during the operation stage. The maintenance and management cost could place a heavy burden on the budget of the bridge owners and managing departments. It is essential to develop effective and efficient bridge inspection and condition assessment approaches to achieve early identification of possible structural damages, deficiencies, and other anomalies. This would help with timely implementation of measures to enable maintenance and repair at an early stage, prevent continuous damage accumulation, sudden failure or even fatal collapse, and maximize the bridge lifespan with lower life-cycle costs [1,5].

Over the last thirty years, structural health monitoring (SHM) technologies have been thoroughly investigated and have been in constant development for bridges [6,7]. SHM systems have been deployed on a large number of long-span bridges all over the world to monitor and track the structural responses and external excitations, such as environmental factors, traffic loads, extreme events including earthquakes and typhoons, etc., to detect structural anomalies and deterioration, and identify damage for decision making. SHM has been a great tool for effectively evaluating bridge performance and health state, assisting bridge owners and managing departments for better bridge maintenance and management, and ensuring the functionality, serviceability, and safety of bridges during the operational stage [8,9].

To achieve the goals and objectives of SHM, various advanced sensing technologies are employed with interdisciplinary knowledge to support SHM with convenient monitoring approaches and reliable data acquisition. Displacement measurement has been one of the objectives of SHM as displacement serves as a critical metric for condition assessment and performance evaluation [10]. By using displacement data, the key performance indicators (KPI) that reflect structural behaviors and characteristics can be extracted, such as bridge lateral and longitudinal load distributions, unit influence line, deflection profile, and modal properties [11]. However, displacement monitoring of large-scale structures is still a challenging task in current research studies and engineering practices of structural health monitoring.

There are two main types of displacement measurement methods: (1) contact-type methods, and (2) non-contact-type methods. Contact-type methods, such as linear variable differential transformers (LVDT), potentiometers, magnetostrictive displacement sensors, and dial gauges can output accurate measurement results, but they need static reference to fix the measurement base close to the measurement targets, which is difficult for large structures. Other contact-type methods, such as using double integration of recorded acceleration data or displacement derivation from the strain–curvature–deflection relationship, can be applied to large structures, but a series of complicated filtering steps, curve fitting, and assumptions based on structures are involved to remove noise and the accuracy requirement may not be satisfied. Another challenge of contact-type methods is that the tedious work involved, such as sensor installation, cable wiring, and data acquisition instrumentation, is time-consuming and costly. Examples of non-contact-type methods include global positioning systems (GPS), laser doppler vibrometers (LDV), radar interferometry, and total station. The non-contact-type methods do not require access to the measured structures, which saves time and effort for monitoring arrangements. However, the prices of the devices for these technologies are very expensive, especially when real-time continuous monitoring is required [12].

In recent years, extracting structural displacement from images by using computer vision-based algorithms has become a hot research topic in the field of civil structural engineering [11,13,14,15,16]. The advantages of computer vision-based structural monitoring, such as being non-contact, low cost, and less time-consuming, have attracted increasing attention from the community of SHM, and computer vision-based SHM methods provide alternative solutions for conventional SHM [17,18,19,20]. Various computer vision-based algorithms have been developed and case studies have been conducted on different types of structures including small-scale structures in the laboratory [21,22,23], and real-life structures such as footbridges [24], short–mid-span highway bridges and railway bridges [25,26], buildings [27], grandstands and stadiums [28], and cable or line structures [29,30]. Although there is a large number of experimental cases of computer vision-based displacement measurement on civil infrastructures, for real-life structures with large sizes, especially the landmark long-span bridges, few studies have been published. Brownjohn et al. (2017) [31] tested the computer vision-based displacement monitoring system on the Humber Bridge in the United Kingdom, which has a main span of 1410 m. Luo and Feng (2018) [32], and Feng and Feng (2017) [33] conducted experiments on Manhattan Bridge in New York, which has a main span of 451 m, to verify their proposed computer vision displacement measurement algorithms. Wahbeh et al. (2003) [34] measured the displacements of the Vincent Thomas Bridge in California, which has a main span of 457 m, by using image-processing methods and two red lights. By employing a consumer-grade digital camera and area-based template matching and Lucas–Kanade sparse optical flow algorithms, Bocian et al. [35] measured the dynamic responses and extracted the modal damping and frequencies of a cable-stayed bridge that had a central cable-stayed section of 612 m. For long-span bridge applications, the measurement distances, i.e., the distance between the camera and the targeted locations on bridges, are generally much larger than laboratory experiments or close-range applications on real-life structures. The measurement distances of the above-mentioned examples are over hundreds of meters. The long-distance application scenarios make it difficult for the computer vision-based methods to ensure similar measurement accuracy compared to laboratory experiments or close-range applications, considering the possible adverse environmental factors such as airflow uncertainties, wind effects, and anomalous light changes. It is valuable to demonstrate the application of landmark long-span bridges, especially for bridges with a main span over 1000 m, and draw the attention of the SHM community with the challenges and considerations involved in the engineering practices of computer vision-based monitoring on long-span bridges.

### 1.2. Objectives and Scope

**Figure 1:**
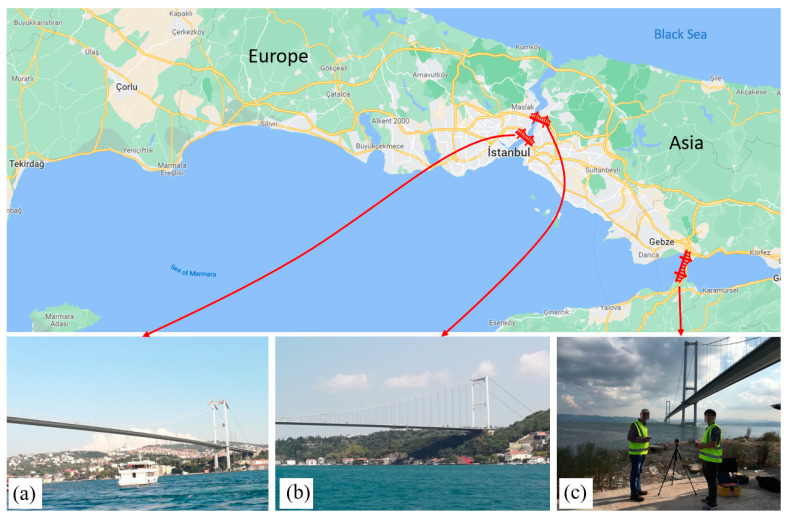
An overview of the tested long-span bridges in Turkey: (**a**) the First Bosphorus Bridge, (**b**) the Second Bosphorus Bridge, and (**c**) the Osman Gazi Bridge.

This paper presents three unique studies of computer vision-based structural displacement monitoring of long-span bridges in Turkey. The three long-span bridges are (a) the First Bosphorus Bridge (also called the 15 July Martyrs Bridge), (b) the Second Bosphorus Bridge (also called the Fatih Sultan Mehmet Bridge), and (3) the Osman Gazi Bridge, as shown in Figure 1. All the three long-span bridges are landmark bridges and are among the largest bridges in the world. The First and Second Bosphorus Bridges connect two continents (Europe and Asia), with main spans of 1074 m and 1090 m, respectively. The Osman Gazi Bridge is the seventh longest bridge in the world, with a main span of 1550 m. The three bridges are suspension-cable-supported structures, with main spans over 1000 m. More details of the three landmark bridges can be found in the following sections. The presented studies aim to investigate the global behavior of bridge deck by using computer vision-based structural monitoring, because deck is one of the major components of bridges, directly carrying the traffic loads and transferring the loads to hangers in suspension bridges. In addition, major permanent behavioral changes due to extreme events such as earthquakes and hurricanes can be observed on the bridge decks. In this study, the concepts and methods for computer-vision SHM for long-span bridges are presented first. Results from computer vision-based measurements are compared with those obtained from finite element (FE) analyses and the existing literature on the First and Second Bosphorus Bridges, and field experiments using accelerometers on the Osman Gazi Bridge conducted by the authors. This paper also summarizes the considerations of computer vision-based structural monitoring over long measurement distances, particularly for long-span bridges.

## 2. Computer Vision-Based Displacement Measurement Using Zero-Mean Normalized Cross-Correlation and Homography Transformation

**Figure 2:**
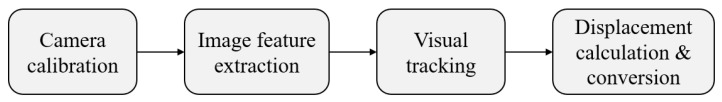
Flowchart of computer vision-based displacement measurement method.

The idea of the computer vision-based displacement measurement method is to use computer vision to track the motions of selected monitoring targets from the video or image sequences recorded by cameras, calculate the location changes of the targets, and convert the location changes in pixel units to displacements in physical units by using the camera calibration information [11]. Figure 2 shows a flowchart of the computer vision-based displacement measurement method using feature matching. Generally, there are four steps in the procedure of extracting displacement using computer vision. First, camera calibration is conducted to determine the relationship between the camera and the real world. Second, image features are selected and extracted for visual tracking. In this step, the measurement targets are also selected in the image. Third, visual tracking is performed in the recorded video/image sequences to obtain the motion of the targets. Finally, the displacements of the targets in images are extracted from the visual tracking results by calculating the location changes of measurement targets in pixel units in images. The actual displacements of measurement targets in the real world can be obtained by converting the displacement in pixel units in images to the physical units such as millimeters in the real world with camera calibration information.

**Figure 3:**
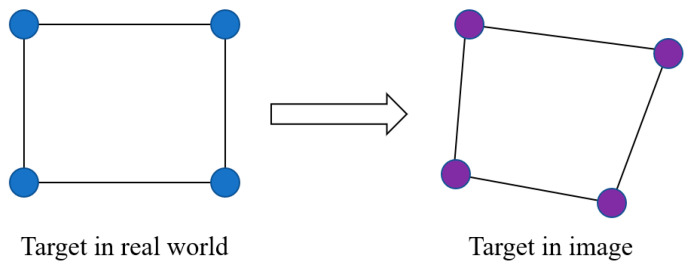
Image projection using the planar homography matrix and four point correspondences.

There are three main approaches to conducting camera calibration for displacement measurement purposes: (1) scale factor, (2) full projection matrix, and (3) planar homography matrix. The scale factor method is convenient and easy to use, especially when the camera axis is perpendicular to the target plane. When there is an angle between the camera axis and target plane, the scale factor has to be revised by using geometric calculations. More details can be found in Reference [14] and Reference [36]. Full projection matrix is a detailed camera calibration method which is required to conduct a series of tests to obtain the intrinsic and extrinsic parameters for the full projective transformation between the image and the real world. It is a comprehensive way to find the relationship in 3D between the image and the real world, but it may not be convenient for displacement measurement in field application, especially for the long-span bridge application with the measurement distances over hundreds of meters. The planar homography matrix is a practical way to build the connection between image and real world, especially for displacement measurement of civil structures where the monitored targets generally move within a plane. The planar homography transformation is a degraded version of full projective transformation from 3D to 2D. The planar homography matrix method also considers the angle problems when the camera axis is not perpendicular to the target plane. As shown in Figure 3, a rectangular target, marked by four blue points at its vertices in the real world (left side), is projected onto the image (right side). Due to the different plane orientations between the real rectangle and the camera, the target’s shape appears distorted in the image, no longer maintaining its rectangular form. This altered shape is denoted by the purple points in the image. This projective change can be presented by the planar homography matrix, **H**. The mapping from the target in image to real world can be built by multiplying the matrix **H** on the left of the points of the target in the image. To obtain the **H** matrix for the mapping, at least four point correspondences are required. The detailed derivation with equations can be found in reference [11]. For the case studies of long-span bridges presented in this paper, the manual targets and bridge guardrails with known dimensions can be used to build the planar homography matrix.

Visual tracking is a core step in the procedure of computer vision-based displacement measurement. As detailed in Reference [11], a plethora of algorithms—including cross-correlation-based template matching, edge detection, feature matching, geometry matching, color-based tracking, full field optical flow estimation, optical flow estimation at feature points, and deep learning-based object tracking—can be implemented to determine the target motion. Among these methods, cross-correlation-based template matching methods are the most commonly used for displacement measurement and they utilize the low-level image features, such as grayscale intensities or gradients, to serve as the basis for visual tracking. Cross-correlation-based template matching does not necessarily need customized geometric shapes for geometry matching methods, sufficient image details to extract feature points or corners in feature matching and optical flow estimation with feature point methods, or complicated image-processing operations and steps required in more advanced methods.

For the applications of the long-span bridge measurements in long distance, the zero-mean normalized cross-correlation (ZNCC) is implemented to serve visual tracking purposes, and raw image grayscale intensities are selected as features in template. More details about ZNCC can be found in reference [11].

## 3. Experiment on the First Bosphorus Bridge

### 3.1. General Features of the First Bosphorus Bridge

The First Bosphorus Bridge is the first bridge that was built in Istanbul to cross the Bosphorus Strait and connect Europe and Asia. The construction was completed in 1973 and it was the longest suspension bridge in Europe and the fourth longest bridge in the world at that time. At present, this bridge still provides a significant link between two continents in the transportation system of Istanbul’s metropolitan area [3,4]. An overview picture and the location of the First Bosphorus Bridge are shown in Figure 1a. The First Bosphorus Bridge is a steel suspension bridge with three standard traffic lanes. It has a similar structural type and design considerations to those of the Severn and Humber Bridges, which are the first modern bridges in Europe with an aerodynamic box deck section. The First Bosphorus Bridge has a main span length of 1074 m and two approach spans with lengths of 231 m at the European side and 255 m at the Asian side. It is a gravity-anchored long-span suspension bridge, and only the main span is suspended with hangers and the approach spans are supported at the base with columns of various heights. The original hangers were inclined, and in 2015, they were replaced by vertical hangers [37]. The elevation of the towers has a height of 165 m from sea level to saddle [4]. The main traffic types of the First Bosphorus Bridge are cars and buses, while commercial vehicles such as trucks cross the Second Bosphorus Bridge.

### 3.2. Experimental Setup

**Figure 4:**
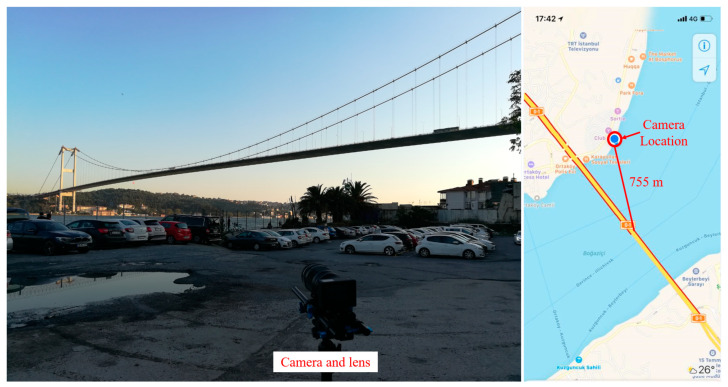
Experimental setup of the First Bosphorus Bridge.

Figure 4 shows the experimental setup of the computer vision-based structural displacement monitoring of the First Bosphorus Bridge. The experiment was conducted during the afternoon of 15 September 2018, at local time. A camera (Z camera E1, Z CAM, Shenzhen, China) with a resolution of 4K (3840 × 2160 pixels), a frame rate of 30 frames per second (FPS), and a 75–300 mm zoom lens (Olympus M.Zuiko ED 75-300 II, Olympus Corporation, Breiningsville, PA, United States) was set up on the European side, north of the bridge. The distance between the camera and the bridge’s midspan is roughly 755 m, as measured using Google Maps.

**Figure 5:**
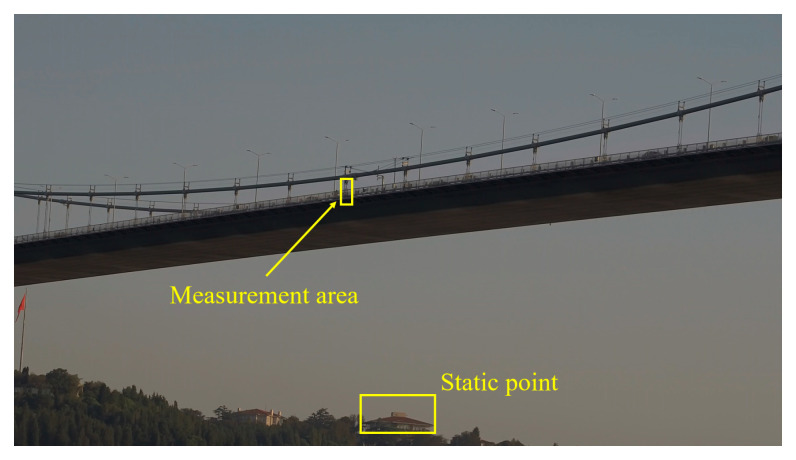
Measurement point and static point on the background.

Figure 5 shows the captured image from the camera for displacement measurement. The guardrail and edges of the deck on the midspan of the northern side of the bridge was selected as the tracking target (marked as measurement area) as shown in Figure 5. During the experiment, the wind caused the camera to shake throughout. According to data from a nearby weather station (Sariyer, TU) [38], the maximum sustained wind speed was 1.8 km/h and gust was 15 km/h, as a general reference for the wind conditions. The wind and gust at the camera’s location might be larger than that at the weather station. A building in the background was regarded as a static point of reference and was selected to mitigate the camera motion problem by using background subtraction. The experiment was conducted on a Saturday afternoon, and at that time (around 5 p.m. to 6 p.m.), the traffic was becoming very busy, and the light was decreasing. Tracking the guardrails over a long distance was quite difficult.

### 3.3. Results Analysis and Verification

**Figure 6:**
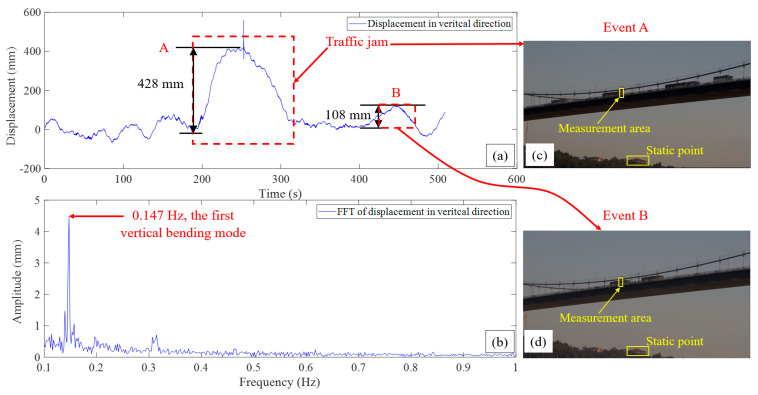
Displacement results of the midspan of the First Bosphorus Bridge: (**a**) vertical displacement time period at midspan, (**b**) FFT of displacement in vertical direction, (**c**) image of the traffic event A related to the displacement measurement, (**d**) image of the traffic event B related to the displacement measurement.

As shown in Figure 6, in the first 180 s of the displacement time period, the maximum displacement was around 100 mm, a traffic jam occurred between t = 200 s and t = 300 s, and the vehicles began to accumulate, slowly moving and even stopping at times on the bridge. In this traffic jam, marked as traffic event A in the red dashed box in Figure 6, a large deflection of around 428 mm was induced, while this deflection was much less than the commonly considered limit defined by *L*/800. *L* is the length of the span. It should be noted that the *L*/800 metric, in many cases a rule of thumb, is mainly used for highway bridges, which are typically stiffer. The metric used here has a general comparative evaluation.

Traffic event A observed from the displacement time period in Figure 6a can be seen from the captured image as shown in Figure 6c. From 260 s, the traffic flow was decreasing, and the deflection started to decrease to the normal level. Around 450 s, as shown in Figure 6d, there were two big buses at the midspan which also induced a large deflection (108 mm), marked as traffic event B in Figure 6a.

**Figure 7:**
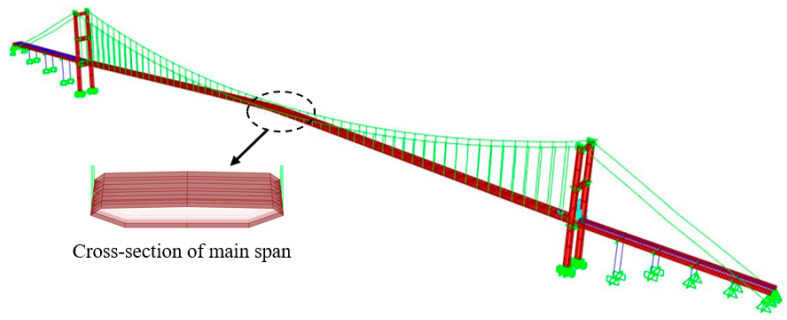
An overview of the 3D FE model of the First Bosphorus Bridge.

In this experiment, there was no direct comparison of the displacement measurement with other types of displacement sensors. To verify the feasibility for the applied computer vision-based displacement measurement system, a 3D FE model was developed in SAP2000, and the FE model was based on the as-built drawings and the most updated status after the hanger replacement, as shown in Figure 7. The deck of the main span, the tower, the portal beams, and the approach viaduct deck consisting of a steel box girder and hand-built steel cross I-beams were modelled with shell elements. The approach viaduct columns with circular box section were modelled with frame (beam) element. The cable elements, hanger, back-stay, and main cables are developed by the cable properties, including sag effect. The developed FE model was verified with SHM data and the experimental results presented in reference [39], which were from accelerometers. More details of the 3D FE model of the bridge can be found in the previous work [37].

Given the difficulties in accessing the bridge’s surface and obtaining real traffic conditions and vehicle load distributions during our experiments, it is challenging to accurately simulate the exact traffic loads in the FE model. For comparative purposes, we focused on the two specific traffic events depicted in Figure 6c,d, and estimated a set of displacement ranges using the FE model based on the observed traffic conditions from the images captured by the camera.

**Figure 8:**
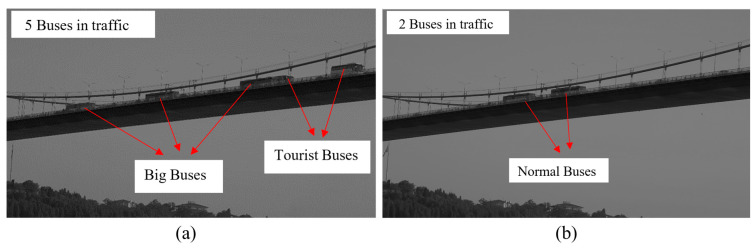
Observed traffic events: (**a**) traffic event A, (**b**) traffic event B.

**Figure 9:**
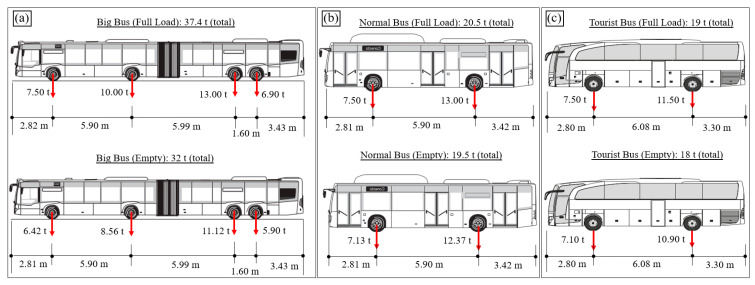
The bus load configurations used for FE analysis: (**a**) big bus with full load and empty status, (**b**) normal bus with full load and empty status, (**c**) tourist bus with full load and empty status.

Figure 8a,b include some notes and markups about the observations, and we made certain estimations of buses and cars based on our observations of the local traffic. We list the three most common bus types and their estimated load configurations in Figure 9, with both fully loaded and empty status: (a) big bus, (b) normal bus, and (c) tourist bus. We also assume that there might be cars in other lanes, and we use compact cars with 1.2 tons to represent the assumptions. In traffic event A, as shown in Figure 8a, there were three big buses and two tourist buses, while in traffic event B, as shown in Figure 8b, there were two normal buses. It should be noted that from the images, we can only see the big buses in the lanes towards Europe which were close to the camera side. For the lanes towards Asia, they may be occupied by other large buses which are not visible from the ground-based camera.

**Figure 10:**
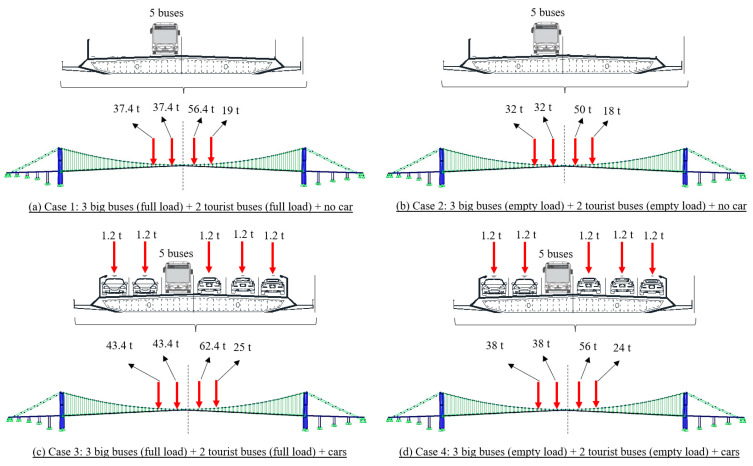
Traffic load configurations for the estimation with FE analysis for traffic event A.

**Figure 11:**
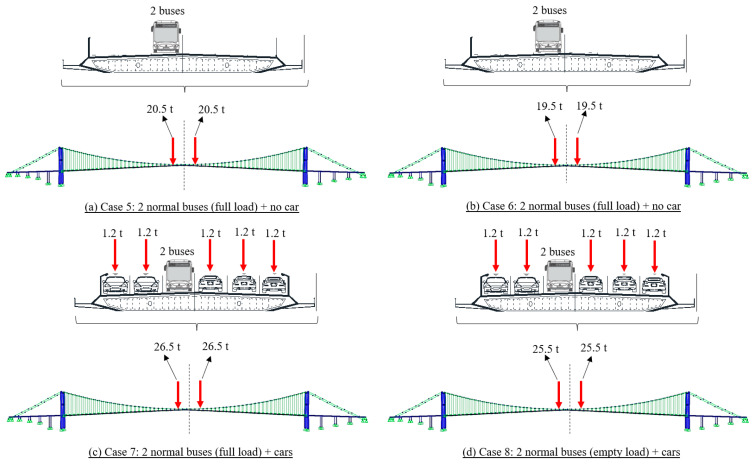
Traffic load configurations for the estimation with FE analysis for traffic event B.

For the approximation of the possible traffic conditions, we assumed four different cases for traffic events A and B, as shown in Figure 10 and Figure 11, respectively. Taking traffic event A as an example, Case 1 and Case 2 represent the cases that only buses occupied the lane but with full load and empty status, respectively. In Case 3 and Case 4, in addition to the buses set in Case 1 and Case 2, small size cars are used to occupy the lanes in the same cross-section with the buses. The traffic configurations of traffic event B, Cases 5 to 8, are similar to the settings of Cases 1 to 4 of traffic event A. The multi-axle load of each vehicle was combined as a concentrated load, considering that the length of the vehicle is much smaller than the length of the bridge span. Additionally, only static analysis was conducted at the time of the traffic jam because traffic was stop-and-go during the data collection period.

With the verified 3D FE model and the assumed traffic configurations based on the observations from the captured images, the displacement comparisons between the computer vision-based method and the FE model were obtained. They are summarized in Table 1. It can be seen that the result from computer vision-based measurement, 428 mm, is very close to the estimated range of the displacement results from the FE model for traffic event A, where the range is 211 mm to 408 mm. As a result, the difference in vision-based displacement with the highest load simulated in the FE model is about 4.67%. For traffic event B, the computer vision-based measurement gives the result as 108 mm, which is also close to the estimated range of the displacement calculations from the FE model, from 75 mm to 102 mm. In this case, the difference of vision-based displacement with the highest load is about 5.56%. In the estimated range, the FE model outputs the lower-bound results with the empty bus settings and no car, and the upper-bound results with the fully loaded bus settings and cars. Based on the normal situation for this bridge and the time of data collection, fully loaded bus and car traffic as observed for both A and B is deemed more appropriate. The consistency of loaded conditions for both cases needs to be noted. Although the measurement results from computer vision-based method did not perfectly match with the estimated results from the FE model, it is good enough to demonstrate that the computer vision-based system can give a very close result to the similar magnitude level of the real displacement, considering that the traffic configurations are assumed based on limited information from the captured images. It gives us much confidence in displacement measurement of long-span bridges in long distances using computer vision-based methods. Such methods can be periodically carried out and results can be tracked over the long term to detect any major changes in deflections for similar conditions.

After transforming the time period of vertical displacement into the frequency domain, the vertical frequency of the bridge was found from the frequency spectrum to be 0.147 Hz. Since the measurement data represent a vertical displacement of the midspan, and this is the only peak from the fast Fourier transform (FFT) spectrum, it could be the first vertical symmetric bending mode. The frequency of 0.147 Hz is very close to the first vertical symmetric bending mode reported in the literature [39], 0.149 Hz, which was obtained by using the field measurement data from accelerometers, and the calculated result from the 3D FE model used in this study, 0.150 Hz (about 2% difference with computer vision-based results). There is a lower peak to the left of the first bending mode in Figure 6b, and the frequency is 0.140 Hz. It is hard to recognize this peak as a torsional mode because only the vibration of one side of the bridge was obtained here, and as reported in the literature [39], the frequency of the only torsion mode in the range of 0 Hz to 0.201 Hz is 0.125 Hz. The peak at 0.140 Hz here might be due to the parasitic effects of FFT operations. Overall, the comparisons in the frequency domain verified the accuracy of the displacement measurement of the applied computer vision-based method.

## 4. Experiment on the Second Bosphorus Bridge

### 4.1. General Features of the Second Bosphorus Bridge

The Second Bosphorus Bridge is the second bridge in Istanbul to cross the Bosphorus Strait. The bridge is located on the north side of the First Bosphorus Bridge. The bridge was the 5th longest suspension bridge in the world when it was completed in 1988, and it is now ranked 31st. It is a critical component of the Trans-European Motorway (TEM) in Istanbul, and the bridge is subjected to heavy truck loading. The Second Bosphorus Bridge is also a steel long-span suspension bridge, but the towers of the bridge are supported to the ground level. The ends of the deck are at the level of the tower base and there are no approach spans. The bridge has a main span with a length of 1090 m and two side spans with a length of 210 m. The deck of the main span has an aerodynamic box section with a width and height of 39.40 and 3.00 m, respectively. The main span is supported by the main cable and vertical hangers. The bridge tower has a rectangular box section, and the total height of the bridge tower is 110 m. More details of this bridge can be found in references [3,4].

### 4.2. Experimental Setup

**Figure 12:**
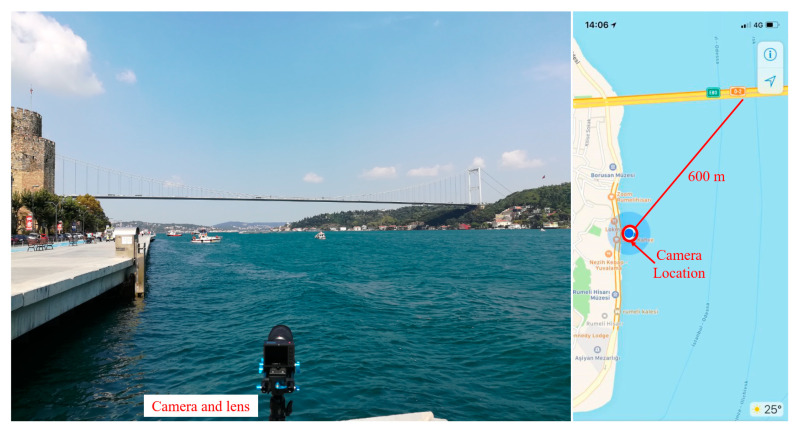
Experimental setup of the Second Bosphorus Bridge.

**Figure 13:**
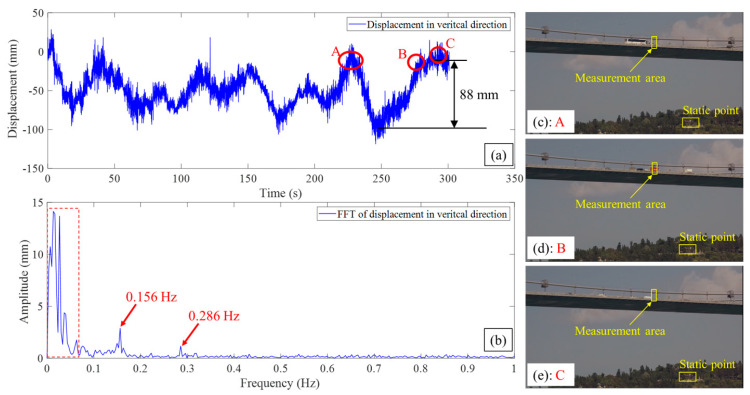
Displacement results of the midspan of the Second Bosphorus Bridge: (**a**) vertical displacement time period at midspan, (**b**) FFT of displacement in vertical direction (red dashed box indicates the low-frequency peaks with the range from 0.006 Hz to 0.064 Hz), (**c**) image of traffic event A related to the displacement measurement, (**d**) image of traffic event B related to the displacement measurement, (**e**) image of traffic event C related to the displacement measurement.

In this experiment, the same camera and lens used in the First Bosphorus Bridge experiment were used to monitor the midspan displacement of the Second Bosphorus Bridge. As shown in Figure 12, the camera and lens utilized in the experiment of the First Bosphorus Bridge were set up on the European side and southern side of the bridge. The distance from the midspan to the camera is around 600 m, as measured using Google Maps. The experiment was conducted at around 2 p.m. local time, on a Saturday afternoon, 15 September 2018. As shown in Figure 13, the guardrails and edges of the midspan were selected as tracking target (marked as measurement area), and a building in the background was selected as the static point to illuminate the camera motion caused by wind effects.

### 4.3. Results Analysis and Verification

Figure 13 shows the displacement results of the mid-span of the Second Bosphorus Bridge. The largest displacement in the time period is 88 mm. There was no traffic jam during this monitoring. Three traffic events are marked as A, B, and C in Figure 13a, and there were large buses, trucks, and other vehicles crossing the midspan in each marked traffic event in Figure 13c–e, respectively. Compared the deflections with the First Bosphorus Bridge, the deflections here were smaller. The possible reason is that, at the time of the experiment, there were fewer vehicles crossing the bridge. It should be noted that the displacement refers to the change in position of the monitoring target from the reference point. The reference point is established at the beginning of the measurement, specifically at the 0th second. Given this definition, the recorded displacement can be positive, negative, or zero, contingent upon the direction of movement compared to the reference point or the initial condition of the measurement, as shown in Figure 13a.

In the frequency domain analysis, two clear frequencies, 0.156 Hz and 0.286 Hz, were identified from the FFT spectrum, as shown in Figure 13b. Since the measurement data represent a vertical displacement of the midspan, the first frequency, 0.156 Hz, could be the frequency of the first vertical bending mode, which has the maximum variation of 2.56% as given in Table 2. As shown in Figure 13b, there are some low-frequency peaks with the range from 0.006 Hz to 0.064 Hz, as marked in the red dashed box, on the left of the first frequency (0.156 Hz). By validating with the modal information presented in Reference [4], there are no vertical modes in the related range. These peaks might have resulted from the external traffic load occurrences or noises.

**Figure 14:**
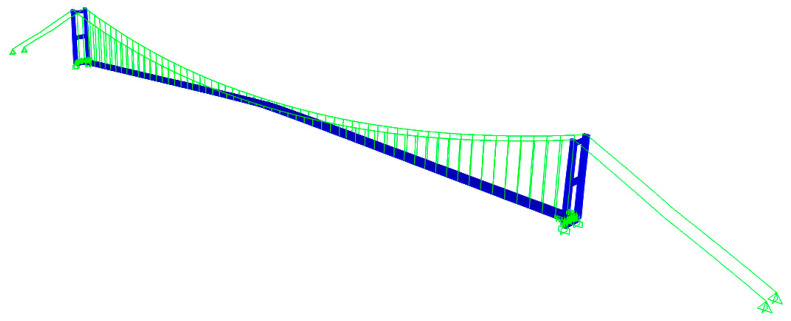
An overview of the spine-beam FE model of the Second Bosphorus Bridge.

Similar to the experiment of the First Bosphorus Bridge, we also did not have a direct comparison with the displacement measurement of other types of displacement sensors. A spine beam FE model of the Second Bosphorus Bridge was developed in SAP2000 as shown in Figure 14, and the FE model was based on the as-built drawings. The deck was modelled as spine beam with frame elements and the towers were also modelled with frame elements. The hanger, back-stay, and main cables were modelled with cable elements, and sag effects were considered. Here, traffic modelling was not considered within the scope of the experiment as this bridge has a more diverse load, and it was not as clear from the distant cameras, and only FE model and SHM-based vibration data were employed for comparison. A very recent experimental study of the Second Bosphorus Bridge by using real data from accelerometers was presented in reference [4], and experimental results from accelerometers in reference [40] are also used for comparative purposes. As can be seen from Table 2, the result from computer vision-based measurement in this study is the same as the result from the real experimental data in the most recently published study [4], and very close to the study conducted years ago [40], and the FE analysis. The comparison gives comparable results and thus provides opportunities for the feasibility of the computer vision-based structural monitoring of long-span bridges over long distances.

## 5. Experiment on the Osman Gazi Bridge

### 5.1. General Features of the Osman Gazi Bridge

The Osman Gazi Bridge is the first long-span suspension bridge constructed for Izmit Bay, and it is at the eastern side of the Marmara Sea, close to the city of Istanbul. The bridge was completed and opened to traffic in 2016, at which time it was the fourth longest suspension bridge in the world. Now, it is ranked as the seventh longest suspension bridge in the world. The bridge has three spans which are 566 m (approach span), 1550 m (main span), and 566 m (approach span). In addition, a transition span with a length of 120 m is connected to each approach span. The approach spans and main span are supported by the main cable and vertical hangers. The bridge tower is built with a box section and with a height of 252 m. The two towers are supported by concrete foundations submerged in the sea. The bridge was designed for the three standard traffic lanes on each side. The deck has an aerodynamic box section with a width of 30.10 m and a height of 4.75 m, braced by the internal truss elements and diaphragm.

For the experiments of computer vision-based measurements, there are two experimental setups based on the locations of the camera: (a) camera on the bank with a distance of 1.35 km, and (b) camera on one of the bridge towers with a distance of 750 m. The two experiments are discussed separately.

### 5.2. First Experiment

#### 5.2.1. Experimental Setup

**Figure 15:**
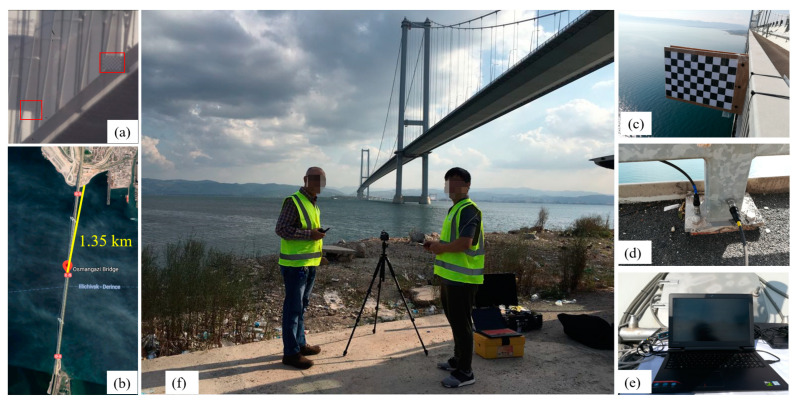
Experimental setup of the first experiment of the Osman Gazi Bridge: (**a**) image from camera (The red boxes, from left to right, indicate the static point and the installed chessboard, respectively), (**b**) camera location in map, (**c**) wooden chessboard, (**d**) accelerometers in vertical and horizontal direction, (**e**) computer for data acquisition, (**f**) camera setup.

As shown in Figure 15, in the first experiment, the camera was located on the bank close to the northern end of the bridge. The distance from the camera to the midspan is around 1.35 km. The 4K camera (Z camera E1) with a Navitar 24× zoom extender lens was employed to measure the displacement of the midspan. Because it was too far from the camera to the midspan of the bridge, it was very difficult to find a target on the bridge to track. A wooden chessboard (967 mm × 676 mm) was installed on the midspan as the tracking target. Two accelerometers were installed on the midspan to measure the vertical and horizontal (transverse) vibrations. Figure 15a shows the images captured by the 4K camera. The image is not clear, the lightness is low, and the boundary is blurred. From the video, it can also be seen that the camera was shaking a lot during the experiment. The left red box in Figure 15a is designed to select the part of the tower as the static refence to illuminate the camera motion problems. The right red box in Figure 15a is the wooden chessboard installed at midspan.

#### 5.2.2. Results Analysis and Verification

**Figure 16:**
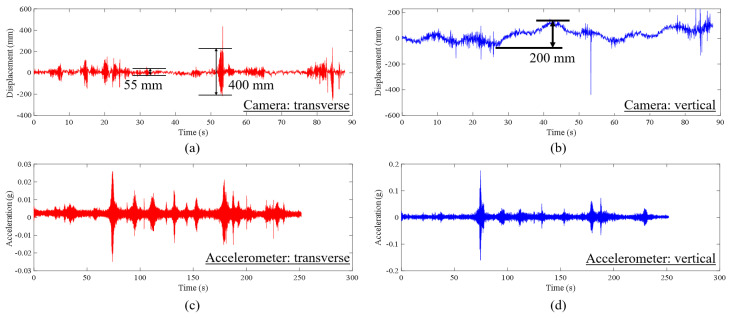
Results of the first experiment on the Osman Gazi Bridge in the time domain: (**a**) transverse displacement from vision-based method, (**b**) vertical displacement from vision-based method, (**c**) transverse acceleration from accelerometer, (**d**) vertical acceleration from accelerometer.

Figure 16 shows the displacement and acceleration results obtained from the camera and accelerometers. From Figure 16a, it can be seen that the maximum displacement in the transverse direction was 400 mm and in the vertical direction was 200 mm. The total durations of the data from camera and accelerometers are around 90 s and 250 s, respectively. The data acquisition system of the accelerometers started early and ended later than the cameras, so that the data duration of the computer vison-based measurement is within the range of the duration of the accelerometers. Direct comparison of the displacement magnitude is not conducted here; instead, only a comparison of the frequency domain is performed.

**Figure 17:**
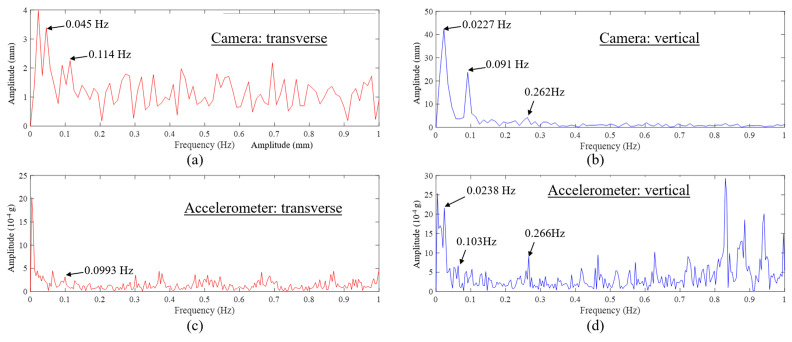
Results of the first experiment on the Osman Gazi Bridge in the frequency domain: (**a**) FFT of transverse displacement from vision-based method, (**b**) FFT of vertical displacement from vision-based method, (**c**) FFT of transverse acceleration from accelerometer, (**d**) FFT of vertical acceleration from accelerometer.

Figure 17 showcases data in the frequency domain. It can be seen from Figure 17a,c that the FFT spectra are marred by noise, making it challenging to discern the transverse vibration modes. It is also worth reporting such data along with possible reasons and recommendations for future work. Nonetheless, when comparing the two spectra, there are some observations: the frequency peak at 0.114 Hz from the camera data aligns with the 0.0993 Hz frequency peak from the accelerometer, reflecting a 14.8% difference. It is also worth highlighting that the two frequencies between 0 Hz and 0.045 Hz evident in the peaks from the camera data in Figure 17a are missing in the results from the accelerometer shown in Figure 17c. Such an omission could likely be attributed to the noise present in the data.

In Figure 17b,d, which depict the spectra of vertical vibrations, the primary peaks in both spectra highlight dynamic frequencies, specifically 0.0227 Hz and 0.0238 Hz, from the camera and accelerometer, respectively. These frequencies are very similar (4.8% difference). However, these low dynamic frequencies may not necessarily represent structural modes. Instead, they could originate from external loads. Additionally, the two dynamic frequencies, 0.091 Hz and 0.262 Hz, captured from the camera data align closely with the frequencies derived from the accelerometers, which are 0.103 Hz and 0.266 Hz, showing differences of 11.6% and 1.5%, respectively. It should be noted that the spectra contain noise, and certain peaks, such as those at 0.262 Hz from the camera and 0.103 Hz from the accelerometer, may not be easily distinguishable. This noise compromises the precision of frequency identification, affecting both vision-based techniques and traditional monitoring methods. One way to minimize such noise is to minimize camera vibration and take measurements at low wind conditions, especially for longer-distance measurements. This might be the case here, especially when transverse vibration spectra are inspected.

### 5.3. Second Experiment

#### 5.3.1. Experimental Setup

**Figure 18:**
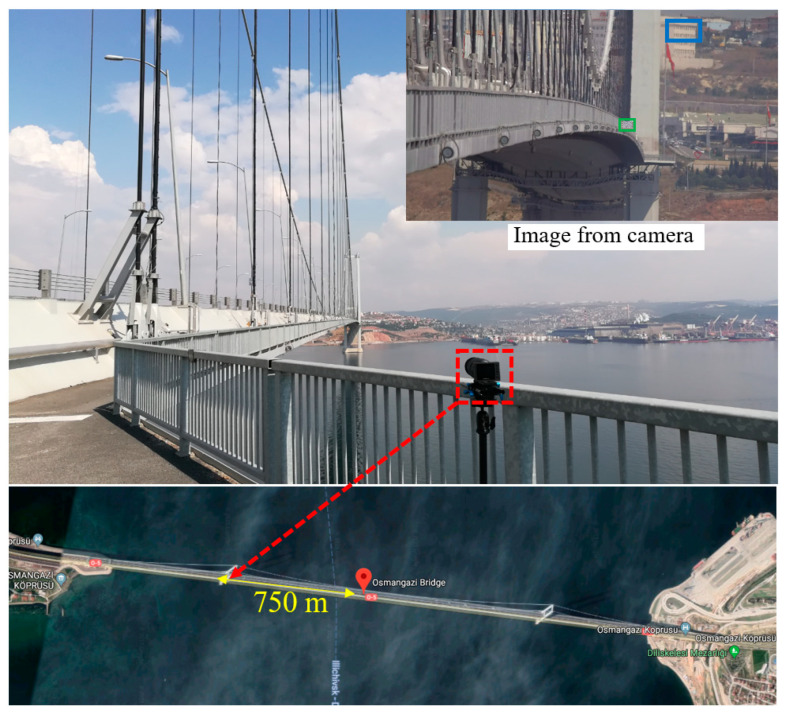
Experimental setup of the second experiment of the Osman Gazi Bridge: the red dashed box indicates the location of the camera.

Figure 18 shows the experimental setup of the second experiment. The camera was located on the tower close to the Izmit side. A 4K camera (Z camera E1) with a 75–300 mm zoom lens (Olympus M.Zuiko ED 75-300 II) was employed to record the video of the motion of the wooden chessboard installed at the midspan. The installed wooden chessboard is marked in the green box as shown in the image from the camera in Figure 18. During the experiment, the camera was vibrated by the wind load. The building in the background marked by the blue box was selected as the static reference to illuminate the camera motion. The distance from the camera to the midspan is 750 m.

#### 5.3.2. Result Analysis and Verification

**Figure 19:**
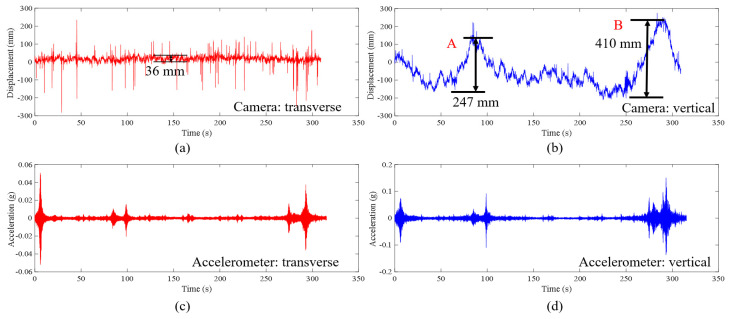
Results of the second experiment on the Osman Gazi Bridge in the time domain: (**a**) transverse displacement from vision-based method, (**b**) vertical displacement from vision-based method, (**c**) transverse acceleration from accelerometer, (**d**) vertical acceleration from accelerometer.

**Figure 20:**
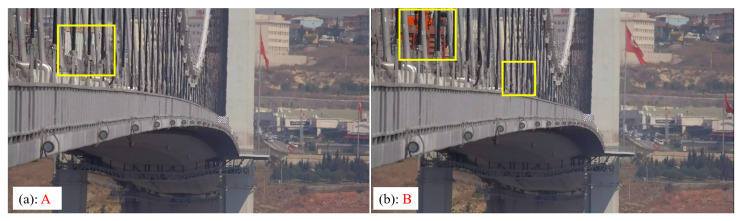
Vehicles on the bridge at time A and B. The yellow boxes indicate the observed traffic events.

Figure 19 shows the results of the second experiment on the Osman Gazi Bridge in the time domain. It can be seen from Figure 19a that the average range of the horizontal displacement is 36 mm. In Figure 19b, at the time points marked as A and B, the displacements are 247 mm and 410 mm, respectively. The large displacement can be identified from the video frames as shown in Figure 20. As shown in Figure 20a, there was a truck crossing the midspan and it induced a displacement of 247 mm; as shown in Figure 20b, there were two trucks crossing the midspan and it induced a displacement of 410 mm.

**Figure 21:**
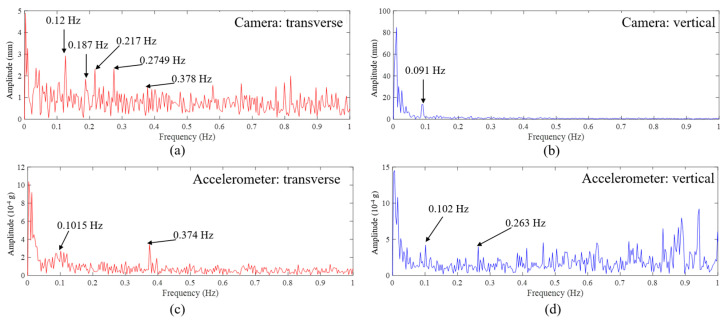
Results of the second experiment on the Osman Gazi Bridge in the frequency domain: (**a**) FFT of transverse displacement from vision-based method, (**b**) FFT of vertical displacement from vision-based method, (**c**) FFT of transverse acceleration from accelerometer, (**d**) FFT of vertical acceleration from accelerometer.

Similar to the observations based on Figure 17, the spectra in Figure 21 exhibit noise, potentially obscuring certain dynamic frequencies. Upon scrutinizing Figure 21a,c, one notices that the initial frequency in the transverse vibration spectrum for the bridge, as captured by the camera, is 0.12 Hz. This is closely aligned with the frequency from the accelerometer, which stands at 0.1015 Hz—a discrepancy of 18%. This frequency is also proximate to the one derived from the initial vision-based monitoring, registering at 0.114 Hz, with a difference of 5.52%.

There are particular peaks, such as those at 0.187 Hz, 0.217 Hz, and 0.2749 Hz in Figure 21a, discerned from the camera data, that remain unidentified in the accelerometer’s results in Figure 21c. Conversely, the prominent peak at 0.374 Hz in Figure 21c from the accelerometer is not observable in the camera’s output as shown in Figure 21a.

As seen in Figure 21b,d, the vibration frequency in vertical direction obtained from the camera data is 0.091 Hz, which is close to the one from the accelerometer obtained at 0.102 Hz (10.8% difference) and also close to the values obtained in the first computer vision experiment (0.091 Hz camera, 0.00% difference), while as shown in Figure 21b, the second mode frequency in the vertical direction is not identified by the data from the camera. It is identified from the acceleration data and is consistent with the results from the first experiment. It should be noted that the results of the dynamic measurements are reasonably matching, while it is difficult to expect a perfect match between the full-contact accelerometers on the surface and computer vision-based data collected at 1350 m and 750 m apart.

## 6. Discussion and Considerations

The unique applications presented in this study achieved non-contact displacement measurements of long-span bridges over distances of several hundreds of meters (600 m, 755 m, and 1.35 km). These provide distinctive results from three applications of computer vision-based structural monitoring on long-span bridges and contrast with most current work that uses computer vision for displacement or dynamic monitoring, which typically focuses on laboratory experiments or close-range applications. Some considerations are summarized as follows:

(1) **Considerations of the verification approaches**: Continuously measuring the structural displacement of large civil structures, particularly long-span bridges crossing rivers, channels, and seas, is a challenging task. Therefore, verifying computer vision-based structural monitoring can be difficult, especially when there is no access to other types of sensors that can directly measure displacement. In this study, verification was conducted using the following two approaches: (a) verifying the monitoring results in the frequency domain using data from both accelerometers and FE models, and (b) verifying displacement magnitude results by estimating a potential range with 3D FE models and observing traffic conditions from images recorded by the camera during displacement measurement experiments. The comparisons yield closely aligned results, providing encouraging evidence to support the use of computer vision-based monitoring from long distances for applications such as long-span bridges.
(2) **Overview of the results**: Displacement and frequency differences between computer vision and FEM results are about 5% and 2%, respectively, for the First Bosphorus Bridge where the camera location is 600 m from the bridge. For the Second Bosphorus Bridge dynamic, the camera distance is 755 m and the difference of sensor and camera-based dynamic results is about 3%. As for the Osman Gazi Bridge, the camera location is 1350 m, and here, we obtain differences for sensor and camera-based frequencies in the order of 1.5% to 18%. The long distance of measurement, possible vibration of the target chess board and camera vibrations, although minimized as much as possible, may explain the relatively high transversal vibration difference. It should also be noted that another camera-based monitoring conducted at Osman Gazi from a 750 m distance showed good consistency with the 1350 m monitoring results.
(3) **Challenges in practice**: The application of computer vision-based monitoring to long-span bridges presents certain challenges due to adverse environmental factors such as airflow uncertainties, camera shaking problems due to wind effects or ground motion, and anomalous light changes, which can result in lower measurement accuracy compared to laboratory experiments and close-range measurements. This issue is particularly pronounced when using extended zoom lenses or lenses with large focal lengths, which limit the light reaching the camera sensors and can result in low-quality images. These challenges eventually would affect the application of long-term monitoring of the computer vision-based system in a continuous fashion. The presented applications mainly focus on short-term monitoring or spot experiments, and the recorded time intervals were within 10 min. Such short-term implementations can be complementary during (e.g., biennial) inspections or for rapid assessment after extreme events. Further solutions need to be investigated to address these challenges to achieve long-term monitoring.
(4) **Possible solutions and considerations**: (a) For long-distance monitoring of long-span bridges, the use of manual targets with distinct geometric patterns or special lights can provide a more practical way to improve measurement accuracy compared to implementing advanced and complicated computer vision-based algorithms. (b) Camera shaking problems caused by wind effects or base vibrations can be mitigated using motion subtraction of reference targets from the background. However, for long-span bridges built in unique areas, finding suitable reference targets can be challenging. (c) Unmanned aerial vehicles (UAVs) can be excellent tools for reducing measurement distances and acquiring high-quality images, offering potential for computer vision-based displacement measurements of long-span bridges, assuming local laws and regulations permit such data collection. However, many long-span bridges are built in relatively open areas that may be exposed to strong wind fields. In these conditions, UAVs can suffer significant wind force, lose balance, or experience large wind-induced vibrations during flights. This presents a challenge for UAV applications on long-span bridges, and additional efforts need to be made to address this issue. Furthermore, the limited battery life of UAVs poses a significant challenge to achieving sustained, long-term monitoring.

## 7. Conclusions

In this paper, the applications of computer vision-based structural monitoring for three unique landmark long-span suspension bridges in Turkey were presented along with an investigation of global structural behavior from vision-based monitoring, FE models, sensor-based monitoring, and other past monitoring studies in a comparative fashion. As a result, the studies presented in this paper validate the measurements using computer vision-based system for long-span bridge applications by using various approaches. This study also discussed the challenges of computer vision-based structural monitoring of large structures. It further provides possible practical solutions and considerations of these challenges.

Overall, the studies on the three unique landmark bridges do not intend to demonstrate that computer vision-based structural monitoring can replace conventional SHM approaches. Instead, it can serve as a complementary approach to conventional SHM. The computer vision-based system can be employed to inspect the permanent SHM systems installed on bridges, intermittently monitor elements not covered by conventional sensors, or rapidly and safely assess structural behavior following extreme events.

## Figures and Tables

**Table 1:** Comparison between the displacement results from computer vision-based method and FE evaluations.

Traffic Event	FE Case No.	FE Case Description	FE Evaluation (mm)	Computer Vision (mm)
A	1	3 big buses (full) + 2 tourist buses (full) + no car	241	428
	2	3 big buses (empty) + 2 tourist buses (empty) + no car	211	
	3	3 big buses (full) + 2 tourist buses (full) + cars	408	
	4	3 big buses (empty) + 2 tourist buses (empty) + cars	379	
B	5	2 normal buses (full) + no car	78	108
	6	2 normal buses (empty) + no car	75	
	7	2 normal buses (full) + cars	102	
	8	2 normal buses (empty) + cars	98	

**Table 2:** Comparison of the results in the frequency domain from computer vision-based method, FE model, and experiments from the literature.

Source	Computer Vision	FE Model	Reference [4]	Reference [40]
Frequency (Hz)	0.156	0.152	0.156	0.155

## Data Availability

The data presented in this study are available on request from the corresponding author.

## References

[1] Fang C., Xu Y.L., Hu R., Huang Z. (2022). A web-based and design-oriented structural health evaluation system for long-span bridges with structural health monitoring system. Struct. Control Health Monit..

[2] Catbas F.N., Susoy M., Frangopol D.M. (2008). Structural health monitoring and reliability estimation: Long span truss bridge application with environmental monitoring data. Eng. Struct..

[3] Bas S., Apaydin N.M., Ilki A., Catbas F.N. (2018). Structural health monitoring system of the long-span bridges in Turkey. Struct. Infrastruct. Eng..

[4] Apaydin N.M., Zulfikar A.C., Cetindemir O. (2022). Structural health monitoring systems of long-span bridges in Turkey and lessons learned from experienced extreme events. J. Civ. Struct. Health Monit..

[5] Yi T.H., Li H.N., Gu M. (2013). Experimental assessment of high-rate GPS receivers for deformation monitoring of bridge. Meas. J. Int. Meas. Confed..

[6] Nicoletti V., Martini R., Carbonari S., Gara F. (2023). Operational Modal Analysis as a Support for the Development of Digital Twin Models of Bridges. Infrastructures.

[7] Innocenzi R.D., Nicoletti V., Arezzo D., Carbonari S., Gara F., Dezi L. (2022). A Good Practice for the Proof Testing of Cable-Stayed Bridges. Appl. Sci..

[8] Deng Y., Ding Y.L., Li A.Q. (2010). Structural condition assessment of long-span suspension bridges using long-term monitoring data. Earthq. Eng. Eng. Vib..

[9] Kankanamge Y., Hu Y., Shao X. (2020). Application of wavelet transform in structural health monitoring. Earthq. Eng. Eng. Vib..

[10] Xu Y., Brownjohn J.M.W., Hester D., Koo K.Y. (2017). Long-span bridges: Enhanced data fusion of GPS displacement and deck accelerations. Eng. Struct..

[11] Dong C.Z., Catbas F.N. (2021). A review of computer vision–based structural health monitoring at local and global levels. Struct. Health Monit..

[12] Huang J., Shao X., Yang F., Zhu J. (2022). Measurement method and recent progress of vision-based deflection measurement of bridges: A technical review. Opt. Eng..

[13] Xu Y., Brownjohn J., Kong D. (2018). A non-contact vision-based system for multipoint displacement monitoring in a cable-stayed footbridge. Struct. Control Health Monit..

[14] Feng D., Feng M.Q. (2018). Computer vision for SHM of civil infrastructure: From dynamic response measurement to damage detection—A review. Eng. Struct..

[15] Spencer B.F., Hoskere V., Narazaki Y. (2019). Advances in computer vision-based civil infrastructure inspection and monitoring. Engineering.

[16] Han Y., Wu G., Feng D. (2022). Vision-based displacement measurement using an unmanned aerial vehicle. Struct. Control Health Monit..

[17] Narazaki Y., Gomez F., Hoskere V., Smith M.D., Spencer B.F. (2021). Efficient development of vision-based dense three-dimensional displacement measurement algorithms using physics-based graphics models. Struct. Health Monit..

[18] Kong X., Li J. (2018). Vision-based fatigue crack detection of steel structures using video feature tracking. Comput. Civ. Infrastruct. Eng..

[19] Shao Y., Li L., Li J., An S., Hao H. (2021). Computer vision based target-free 3D vibration displacement measurement of structures. Eng. Struct..

[20] Ribeiro D., Santos R., Cabral R., Saramago G., Montenegro P., Carvalho H., Correia J., Calçada R. (2021). Non-contact structural displacement measurement using Unmanned Aerial Vehicles and video-based systems. Mech. Syst. Signal Process..

[21] Gomez F., Narazaki Y., Hoskere V., Spencer B.F., Smith M.D. (2022). Bayesian inference of dense structural response using vision-based measurements. Eng. Struct..

[22] Jiao J., Guo J., Fujita K., Takewaki I. (2021). Displacement measurement and nonlinear structural system identification: A vision-based approach with camera motion correction using planar structures. Struct. Control Health Monit..

[23] Kromanis R., Kripakaran P. (2021). A multiple camera position approach for accurate displacement measurement using computer vision. J. Civ. Struct. Health Monit..

[24] Wang M., Ao W.K., Bownjohn J., Xu F. (2022). A novel gradient-based matching via voting technique for vision-based structural displacement measurement. Mech. Syst. Signal Process..

[25] Tian L., Zhang X., Pan B. (2021). Cost-Effective and Ultraportable Smartphone-Based Vision System for Structural Deflection Monitoring. J. Sens..

[26] Dong C.Z., Bas S., Catbas F.N. (2020). A portable monitoring approach using cameras and computer vision for bridge load rating in smart cities. J. Civ. Struct. Health Monit..

[27] Wang J., Zhao J., Liu Y., Shan J. (2021). Vision-based displacement and joint rotation tracking of frame structure using feature mix with single consumer-grade camera. Struct. Control Health Monit..

[28] Dong C.Z., Celik O., Catbas F.N. (2019). Marker free monitoring of the grandstand structures and modal identification using computer vision methods. Struct. Health Monit..

[29] Jana D., Nagarajaiah S. (2021). Computer vision-based real-time cable tension estimation in Dubrovnik cable-stayed bridge using moving handheld video camera. Struct. Control Health Monit..

[30] Jiang T., Frøseth G.T., Rønnquist A., Fagerholt E. (2020). A robust line-tracking photogrammetry method for uplift measurements of railway catenary systems in noisy backgrounds. Mech. Syst. Signal Process..

[31] Brownjohn J.M.W., Xu Y., Hester D. (2017). Vision-based bridge deformation monitoring. Front. Built Environ..

[32] Luo L., Feng M.Q. (2018). Edge-Enhanced Matching for Gradient-Based Computer Vision Displacement Measurement. Comput. Civ. Infrastruct. Eng..

[33] Feng D., Feng M.Q. (2017). Experimental validation of cost-effective vision-based structural health monitoring. Mech. Syst. Signal Process..

[34] Wahbeh A.M., Caffrey J.P., Masri S.F. (2003). A vision-based approach for the direct measurement of displacements in vibrating systems. Smart Mater. Struct..

[35] Bocian M., Nikitas N., Kalybek M., Kużawa M., Hawryszków P., Bień J., Onysyk J., Biliszczuk J. (2023). Dynamic performance verification of the Rędziński Bridge using portable camera-based vibration monitoring systems. Arch. Civ. Mech. Eng..

[36] Xu Y., Brownjohn J.M.W. (2018). Review of machine-vision based methodologies for displacement measurement in civil structures. J. Civ. Struct. Health Monit..

[37] Bas S., Dong C.Z., Apaydin N.M., Ilki A., Catbas F.N. (2020). Hanger replacement influence on seismic response of suspension bridges: Implementation to the Bosphorus Bridge subjected to multi-support excitation. Earthq. Eng. Struct. Dyn..

[38] Visual Crossing Corporation Historical Weather Data for Kaffrine. https://www.visualcrossing.com/weather-history/41.133,29.067/metric/2018-09-15/2018-09-15.

[39] Soyoz S., Dikmen U., Apaydin N., Kaynardag K., Aytulun E., Senkardasler O., Catbas N., Lus H., Safak E., Erdik M. (2017). System identification of Bogazici suspension bridge during hanger replacement. Procedia Eng..

[40] Brownjohn J.M.W., Dumanoglu A.A., Severn R.T. Full-Scale Dynamic Testing of the 2nd Bosporus Suspension Bridge. Proceedings of the 10th World Conference Earthquake Engineering.

